# Hemodynamics under General Anesthesia in Glenn/Fontan Circulation?

**DOI:** 10.1007/s00246-021-02559-4

**Published:** 2021-02-07

**Authors:** Dietmar Schranz

**Affiliations:** Pediatric Heart Center, Johann Wolfgang Goethe University, Theodor Stern Kai 6, 60385 Frankfurt, Germany

Letter to the Editor

Regarding the article of Sanchitha Guruchandrasekar et al. [1]. With high interest, I read the article on predictive hemodynamic data of patients with single ventricle (SV) physiology prior to Fontan completion. However, my attention turned immediately to the Methods under which the hemodynamic data were obtained. The authors described that over a ten-year era, the hemodynamics were obtained by cardiac catheterization under general anesthesia with intubated and controlled ventilation. I have no doubt concerning the author’s findings, but absolute calculated vascular resistance and CI numbers achieved under such catheter conditions evoke following questions:

1. Why complex calculations (CI, PVRI, SVRI) in a complex circulation is believed to be needed? Although it is known, that it already contains calculation errors. Why do the authors not base their decisions on pure measured raw data as pressures and oxygen saturations?
2. Why are the examinations carried out under general anesthesia, although the hemodynamics of the “passive” pulmonary circulation (Shunt-, Glenn-connection) is significantly influenced, which is distinct following complete Fontan, but is in no way comparable to “real” life under spontaneous breathing at rest.
3. Therefore, in the light that NEEP (negative endexspired pressure = spontaneous breathing) is in either case better for pulmonary and consecutively systemic blood flow than PEEP (= ventilation) in a (hemi-) Fontan circulation, the methods of the electively intubated positive pressure ventilated patients should present some settings at least the mean airway pressures during controlled ventilation and the FiO_2_ level to retrace the calculated measurements and in particular the pulmonary artery pressure, inferior caval pressure both in relation to the systemic arterial pressures and saturations as well?
4. Last but not least, would it be interesting to know how the decision for general anesthesia is argued or whether it is based on the anesthesiologic preference?

It remains a mystery to me why it is the rule and not the exception in some facilities for patients with SV-pathophysiology to perform particularly diagnostic cardiac catheterizations under general anesthesia? Experienced doctors/anesthesiologists are easily able to establish a balanced analgo-sedation with a sufficiently spontaneous breathing SV-patient (paCO_2_ 40–45 mmHg). General anesthesia in infant according to Norwood or Hybrid stage-I (S1P) can be dangerous because systemic blood flow is impaired by a pulmonary run-off and according to S2P (Glenn) or S3P (Fontan) due to a compromised pulmonary blood flow with possible problems achieving a sufficient preload for the SV.
